# Mapping Actuarial Criteria for Parkinson’s Disease-Mild Cognitive Impairment onto Data-Driven Cognitive Phenotypes

**DOI:** 10.3390/brainsci12010054

**Published:** 2021-12-30

**Authors:** Lauren E. Kenney, Adrianna M. Ratajska, Francesca V. Lopez, Catherine C. Price, Melissa J. Armstrong, Dawn Bowers

**Affiliations:** 1 Department of Clinical and Health Psychology, University of Florida, Gainesville, FL 32603, USA; aratajska@ufl.edu (A.M.R.); flopez1@ufl.edu (F.V.L.); cep23@phhp.ufl.edu (C.C.P.); dawnbowers@phhp.ufl.edu (D.B.); 2 Norman Fixel Institute of Neurological Diseases, University of Florida, Gainesville, FL 32603, USA; Melissa.Armstrong@neurology.ufl.edu; 3 Department of Neurology, University of Florida College of Medicine, Gainesville, FL 32603, USA

**Keywords:** Parkinson’s disease, mild cognitive impairment, movement disorders, cluster analysis, prevalence

## Abstract

Prevalence rates for mild cognitive impairment in Parkinson’s disease (PD-MCI) remain variable, obscuring the diagnosis’ predictive utility of greater dementia risk. A primary factor of this variability is inconsistent operationalization of normative cutoffs for cognitive impairment. We aimed to determine which cutoff was optimal for classifying individuals as PD-MCI by comparing classifications against data-driven PD cognitive phenotypes. Participants with idiopathic PD (*n* = 494; mean age 64.7 ± 9) completed comprehensive neuropsychological testing. Cluster analyses (K-means, Hierarchical) identified cognitive phenotypes using domain-specific composites. PD-MCI criteria were assessed using separate cutoffs (−1, −1.5, −2 SD) on ≥2 tests in a domain. Cutoffs were compared using PD-MCI prevalence rates, MCI subtype frequencies (single/multi-domain, executive function (EF)/non-EF impairment), and validity against the cluster-derived cognitive phenotypes (using chi-square tests/binary logistic regressions). Cluster analyses resulted in similar three-cluster solutions: Cognitively Average (*n* = 154), Low EF (*n* = 227), and Prominent EF/Memory Impairment (*n* = 113). The −1.5 SD cutoff produced the best model of cluster membership (PD-MCI classification accuracy = 87.9%) and resulted in the best alignment between PD-MCI classification and the empirical cognitive profile containing impairments associated with greater dementia risk. Similar to previous Alzheimer’s work, these findings highlight the utility of comparing empirical and actuarial approaches to establish concurrent validity of cognitive impairment in PD.

## 1. Introduction

The experience of Parkinson’s disease (PD) encompasses not only the prototypical motor symptoms but also a plethora of non-motor symptoms including cognitive changes. Past research estimates that approximately 40% of people with PD have mild cognitive impairment (PD-MCI) at any given time, and up to 80% of individuals with PD will develop dementia after living with the disease for 15–20 years [1,2]. However, the trajectory of cognitive changes can differ among individuals with clear diagnoses of idiopathic PD—with some declining more rapidly than others [3]. Therefore, while the endpoint of the trajectory is known for many individuals with PD, the question remains who is most at risk for a more rapid transition to Parkinson’s disease dementia (PDD).

Two lines of research aim to answer this question. Some studies take an empirical approach by statistically examining neuropsychological data to see what patterns of cognitive performance arise. This is often done via use of cluster analytic techniques, resulting in distinct clusters or cognitive phenotypes. Others take an a priori classification approach, meaning that mild cognitive impairment (MCI) is designated by specific impairment criteria, which are then used to identify patterns of deficits across cognitive tests or domains. One way of doing this is via “actuarial classification criteria”, defined as using objective, pre-established numerical definitions of impairment, rather than a consensus diagnosis or clinical judgment. Both empirical (cluster analytic) and actuarial/clinical theoretical classification approaches aim to characterize distinct cognitive profiles in PD-with the hope of subsequently determining if certain cognitive profiles or subtypes connote greater risk of developing PDD at faster rates.

Recently, comparison of the predictive utility of these two types of approaches (cluster analysis vs. a priori classification) has gained traction among researchers in the MCI-Alzheimer’s disease (AD) literature. Indeed, recent studies have found that cognitive phenotypes derived from cluster analyses are more strongly correlated with AD biomarkers and are more strongly linked to dementia progression than traditional a priori classification methods [4,5]. To date, few studies have compared these two approaches in individuals with Parkinson disease or addressed some of the psychometric issues inherent when comparing these approaches to each other [6,7].

Historically, the cognitive sequelae of Parkinson’s disease have been linked to deficits in executive function (e.g., planning, inhibition, problem solving), processing speed, and working memory and attributed to dopaminergic depletion in fronto-striatal networks [8,9]. Even so, various studies have found less prevalent, yet still pronounced, deficits in other cognitive domains such as memory [10,11], visuospatial skills [12,13], and semantic language function [14]. These varying cognitive sequelae of PD play out in both data-driven and classification approaches. Use of data-driven approaches (e.g., cluster analyses) has resulted in some variability in the patterns of PD cognitive phenotypes across studies. Some reveal phenotypes that primarily differ in the level and breadth of cognitive impairments [15,16,17]. Yet, other studies identify clusters that differ in the “types” of cognitive domains that are impaired [18,19,20]. For example, Crowley and colleagues [21], in a recent cluster analytic study, with prospectively recruited individuals with PD, identified three cognitive phenotypes—those showing low executive function, those with low episodic memory performance, and those with no deficits relative to age matched controls.

There is also variability in the rules of the road used by various a priori classification approaches for identifying “mild cognitive impairment” in individuals with PD [22]. Most MCI classification approaches differ in terms of stringency of psychometric criteria such as number of cognitive tests used, use of composite scores, and impairment cutoff criteria. In 2012, the Movement Disorders Society (MDS) published consensus criteria for PD-MCI [23]. The Level II “comprehensive” criteria, which requires more extensive neuropsychological testing beyond a cognitive screener, defined impairment as having two or more tests falling 1–2 standard deviations (SD) below the normative mean *or* demonstrating a relative decline from previous evaluation [23]. While a diagnosis of PD-MCI using the MDS criteria is associated with greater risk of developing PDD [24], even with unified criteria, the prevalence rates of PD-MCI continue to range from 25–65% across studies [25,26]. Such disparate estimates of the portion of individuals with PD-MCI limits this diagnosis’ effectiveness at predicting clinical trajectory to dementia.

In part, the variability in prevalence of PD-MCI across studies results from methodologic differences (i.e., community vs. clinical sample, sample sizes, which neuropsychological tests that are used). However, beyond that, the operationalization of impairment (e.g., use of −1, −1.5 or −2 SDs) is a critical issue. Moreover, variable use of cutoff criteria relates to the notion of “decline” from a previous level, but this hinges on the assumption that test “norms” are inadequate to capture change in certain demographic sectors. Currently, results remain mixed over which cutoff criteria best identifies who is at greatest risk for impending dementia [8,24,27]. Previous work comparing empirical approaches to a PD-MCI classification, based on a prespecified impairment cutoff (−1.5 SD), found greater portions of PD-MCI participants in the more broadly impaired or amnestic phenotypes [6,7].

The overall goal of the present study was to address the issue of “cutoff” criteria head on by comparing clinical classification and data-driven approaches in a large clinical sample of idiopathic PD patients without dementia. We specifically wanted to learn which cutoff was optimal for classifying individuals as PD-MCI. This is important as it works towards establishing more consistent prevalence rates of PD-MCI. To achieve this goal, the current study first examined the influence of using different SD impairment criteria on PD-MCI prevalence rates and subtypes. Next, we identified data-driven cognitive phenotypes using cluster analysis in this same clinical sample. Taken together, these two approaches enabled us to determine how well the PD-MCI classifications mapped onto the cluster-derived cognitive phenotypes using each of three common SD impairment cutoffs.

Based on previous literature [28,29,30,31,32], we predicted that the following empirically based phenotypes would emerge from cluster analyses: normatively average cognition, isolated executive function impairment, and broader cognitive impairment across multiple domains, particularly executive function, memory, and visuospatial. We predicted that a greater proportion of the PD-MCI cases would be represented in the cluster with broad cognitive impairment due to involvement of cortical systems underlying lower memory, visuospatial, and executive performance. Impairments in these domains have previously been shown to put individuals with PD at greater risk of developing PDD [33,34,35]. Finally, we planned to determine which impairment cutoff jointly maximized the model’s sensitivity and specificity and produced the highest classification accuracy.

## 2. Materials and Methods

### 2.1. Design

We performed a cross-sectional, observation study by conducting a retrospective chart review of individuals with PD seen at the University of Florida (UF) Health Norman Fixel Institute for Neurological Diseases. Data encompassed participants’ demographics, disease-related characteristics, neuropsychological assessment, and mood/motivation screening measures.

### 2.2. Participants

Participants included a convenience sample of individuals with idiopathic PD from a large IRB-approved prospectively acquired clinical-research database (INFORM) of movement disorders patients seen at the UF Norman Fixel Institute. For the current study, inclusion criteria were: (1) evaluation between 2002 and 2019 and (2) a diagnosis of idiopathic PD made by a fellowship-trained movement disorders specialist based on the UK Parkinson’s Disease Society Brain Bank Diagnostic Criteria. Exclusion criteria entailed (a) any current major psychiatric disturbance (i.e., unmanaged bipolar disorder, schizophrenia, current episode; *n* = 7); (b) a comorbid essential tremor diagnosis (*n* = 13); (c) previous brain surgery (e.g., deep brain stimulation, pallidotomy; *n* = 87); (d) history of epilepsy, stroke, or brain injury with ongoing cognitive sequela (*n* = 18); (e) missing neuropsychological measures utilized in the study (*n* = 187); (f) evidence of significant cognitive impairment based on scores below 125 on the Dementia Rating Scale-2 (DRS-2, *n* = 127) [36], a cutoff which corresponds to ≤10th percentile [37]. After excluding (*n* = 439) participants from the starting sample (*n* = 933), this resulted in a final N of 494 participants for the current study.

### 2.3. Neuropsychological Measures

All participants received a comprehensive neuropsychological assessment. The battery consisted of the DRS-2 (as a general index of cognitive impairment) and standard neurocognitive measures in the domains of (1) executive function, (2) verbal delayed memory, (3) language, (4) visuospatial skills, and (5) attention/working memory. Specific tests are shown in Table 1, and cognitive measures are grouped by domain based on theoretical considerations [38,39,40]. Norms for each test were derived from test-specific manuals or previously published norms [41] and then converted to *z*-scores. Using normative data allowed us to compare performance to that expected in the population and more closely reflected clinical practice. However, this approach did present the limitation that measures were normed based on different samples and did not all adjust for additional demographics, such as education.

For the majority of cognitive measures, less than 6% of the available sample had missing data. Only the measures included in the visuospatial composite contained a greater portion of missing data (Judgment of Line Orientation: 8.08%, Benton Facial Recognition Test 14.41%). However, when analyzing the pattern of missing values for all cognitive measures, Little’s Missing Completely at Random (MCAR) assumption was supported (χ^2^(304) = 324.61, *p =* 0.20). Because participants needed at least two measures per domain for PD-MCI classification, listwise exclusion (if missing any neuropsychological data) was implemented.

### 2.4. PD-MCI Classification

We classified participants as cognitively normal or meeting actuarial criteria for PD-MCI using three commonly used impairment cutoffs: liberal (−1 SD), midpoint (−1.5 SD), and conservative (−2 SD). For a cognitive domain to be considered impaired, the normative scores on at least two tests within that domain had to fall below the respective cutoff (i.e., −1, −1.5, −2.0 SD). Having at least one impaired domain led to a classification of PD-MCI. This differs from the MDS criteria which allow PD-MCI to be defined by having one impaired test across two separate domains, with the implication that both of those domains are considered impaired. We took a more psychometrically rigorous approach by requiring two or more tests within the same domain to fall below the respective cutoff to assign a classification of PD-MCI. Indeed, this approach is more predictive of PDD [27], minimizes the possibility that poor performance on a single task is an anomaly, and aligns more closely with widespread clinical practice of defining domain impairment based a pattern of deficits across measures within a domain.

Participants designated as having PD-MCI were then divided into subtypes based on whether they were impaired on one or multiple cognitive domains and whether executive function (EF) impairment was present or not. Just as the originally proposed MCI subtypes (amnestic/non-amnestic) aimed to distinguish the presence of or absence of the hallmark characteristic of Alzheimer’s disease [54], we focused on the presence or absence of the most common cognitive impairment (i.e., executive function) in PD. Thus, PD-MCI participants were characterized as being one of four subtypes: single-EF (only EF domain impaired), multi-EF (EF plus at least one other domain impaired), single-non-EF (one domain impaired but not EF), and multi-non-EF (more than one domain impaired but not EF).

### 2.5. Cluster Analyses

For each cognitive domain, a composite score was computed by averaging individual z-scores of tests within a domain. The five domain composite scores were then entered into the cluster analyses to distinguish cognitive phenotypes (groups of participants with similar patterns of cognitive performance). For the current manuscript, we refer to these cluster-derived subtypes as “cognitive phenotypes” to distinguish them from the subtypes derived from the PD-MCI classification. While we had an a priori prediction of three clusters, we tried a range of two to four clusters to ensure ideal data fit; several cluster solutions were generated and contrasted before determining the final cluster structure.

### 2.6. Other Measures

At the time of the neuropsychological evaluation, participants completed self-report screening measures to characterize symptoms of depression (Beck Depression Inventory-II (BDI-II)), apathy (Apathy Scale (AS)), and situational and dispositional anxiety (State-Trait Anxiety Inventory (STAI)) [55,56,57]. To gauge motor symptom severity and disease stage progression, ratings from the Unified Parkinson’s Disease Rating Scale (UPDRS, [58]) Part III and the Hoehn and Yahr scale (H&Y, [59]) were obtained by movement disorder neurologists while participants were “on” their dopaminergic medications. These neurologists also characterized their motor subtype (tremor predominant, akinetic-rigid, or postural instability and gait difficulty). On average, motor symptoms were assessed within 61.33 ± 63.24 days of the neuropsychological evaluation (range = 0–365 days).

### 2.7. Statistics

We used SPSS Version 26 to conduct all the following analyses [60]. We examined demographic variables, clinical characteristics, and cognitive composites for normality and outliers, both visually and statistically. Most variables were not normally distributed—as assessed by histogram inspection, Z-tests of skewness/kurtosis, and Kolmogorov–Smirnov and Shapiro–Wilk normality tests (*p*’s < 0.05). Due to the non-normality of most variables, outliers were defined as scores falling outside 3x the interquartile range. No outliers were detected except for one participant having more years with PD symptoms (54 years) and another having severe depression (BDI = 54). As these two variables were supplementary to our primary aims, all cases were retained within analyses.

Cochran’s Q test compared the PD-MCI prevalence rates using Bonferroni-corrected pairwise comparisons. We independently conducted K-means and Hierarchical cluster analyses (using Ward’s method and squared Euclidean distance) to cross-validate the cluster memberships. To examine the consensus of the two techniques’ cluster memberships, we used cross-tabulations and Pearson chi-square tests of independence.

Using the optimal K-means cluster solution, we compared the derived clusters on demographics, clinical characteristics, and mood/motivation. Due to the clinical nature of our data, some measures were missing, so we used pairwise exclusion for these analyses. Because of the non-normal distribution of these variables, when comparing clusters, we used Kruskal–Wallis H tests (with Bonferroni-corrected pairwise comparisons) for continuous variables and Pearson chi-square tests of independence for categorical variables.

Finally, to quantify the relationship between cluster membership and PD-MCI classification, we used Pearson chi-square tests of independence and binary logistic regressions. Because we aimed to examine the overlap between the prominently impaired cluster and PD-MCI classification, this cluster was used as the reference group in the regression models. Using the models’ sensitivity and specificity, we calculated Youden’s Index values for each cutoff [61]. We then calculated positive and negative predictive values assuming base rates based on the sample’s prevalence rates of PD-MCI and across the range of prevalence rates from previous studies.

## 3. Results

### 3.1. Sample Characteristics

The database search identified 494 individuals meeting inclusion/exclusion criteria. Of these, 338 individuals had neuropsychological assessments performed as part of an evaluation for deep brain stimulation, and 156 individuals had cognitive testing as part of routine clinical care. These groups were largely similar in terms of cognitive performance (see Appendix A Table A1) and thus were treated as a single cohort for the analyses. Participants ranged in age from 38 to 87 years old, with an average age of 64.7 years (Table 2). Participants were well-educated, predominantly male (72%), and white non-Hispanic (94.3%), and had an almost 8-year duration of a PD diagnosis on average. Participants were generally in the early-mid stages of disease severity based the H&Y and the UPDRS Part III. The majority were characterized as the tremor predominant subtype (76.5%), while the rest of the sample were characterized as akinetic-rigid (22.5%) or postural instability and gait difficulty (1.1%). As a group, participants’ DRS-2 total scores were far above the dementia cutoff [37]. The average performance on indices of depression (BDI-II), apathy (AS), and anxiety (STAI) was below clinical cutoff, though there was substantial variability across participants.

### 3.2. Prevalence of PD-MCI and Subtypes

The prevalence of PD-MCI was 40.1% (*n* = 198) when using −1 SD cutoff, 21.5% (*n* = 105) when using the −1.5 cutoff, and 9.1% (*n* = 45) when using the −2 SD cutoff. There was a significant difference between the proportions of PD-MCI classified cases based on cutoff criteria, with a large effect size (χ^2^(2) = 563.10, *p* < 0.001, *η*^2^ = 0.66) and differences in the expected direction (*p*’s < 0.001).

**Figure 1:**
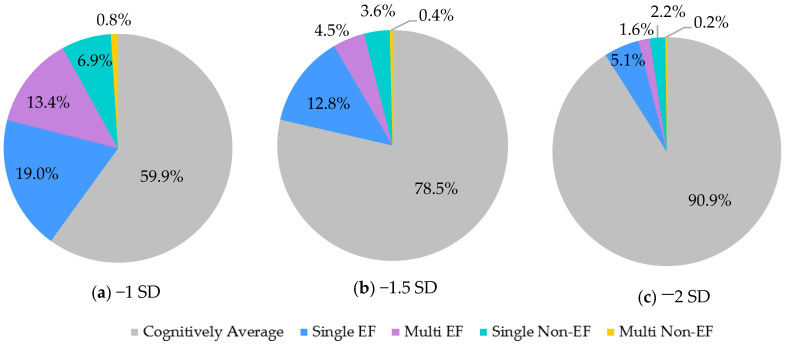
PD-MCI subtype breakdown by actuarial criteria cutoffs. (**a**–**c**) Depict the percentages of the sample that fall into each MCI subtype based on the respective SD cutoff. EF = Executive Function.

Across all three cutoffs, most PD-MCI cases had single-domain impairments (−*1 SD*: 64.59%, −*1.5 SD*: 77.0%, −*2 SD*: 80.22%) (Figure 1). Single-EF was the most common PD-MCI subtype. However, a notable portion of cases fell into non-EF subtypes (−*1 SD*: 19%, −*1.5 SD*: 19%, −*2 SD*: 26%). Classification into these other subtypes was primarily driven by memory and language deficits. Of the PD-MCI cases, when using the −1, −1.5, and −2 SD cutoffs, 33%, 25%, and 31% had memory deficits, and 17%, 15%, and 11% had language deficits, respectively.

### 3.3. Cluster-Derived Cognitive Phenotypes 

**Figure 2:**
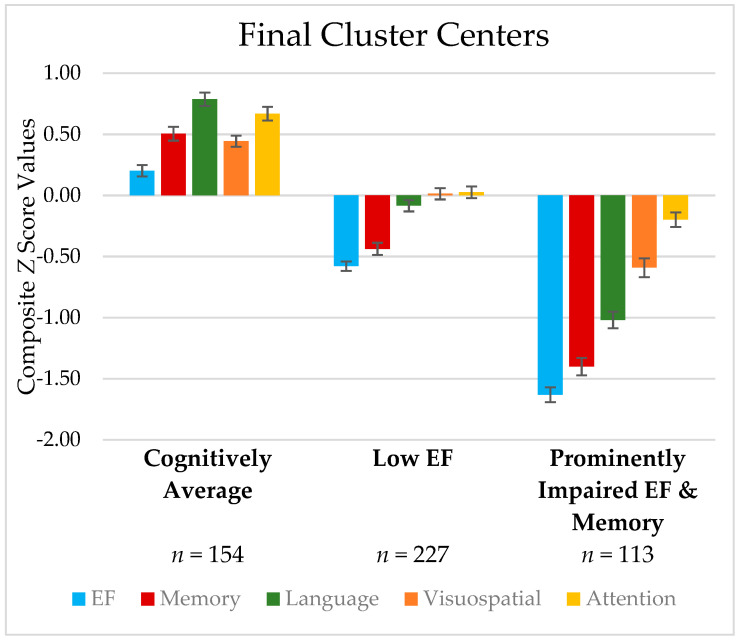
K-Means 3 cluster solution based on pattern of cognitive domain performance. Note. Error bars reflect the standard error of each metric. EF = Executive Function.

Results of the K-means cluster analysis supported the existence of three clusters. Cluster membership was stable after 10 iterations. None of the clusters were unacceptably small. Visual inspection of the cluster centers (Figure 2) revealed a group with average cognition across all domains (*n* = 154), a group with low executive function (Low EF) (*n* = 227), and a group with executive function and memory impairments, as well as low language scores (Prominently Impaired EF/Memory) (*n* = 113).

The hierarchical cluster analysis supported the use of two or three clusters based on the changes in agglomeration coefficients. When examining the proportions of cases assigned to the same cluster by both clustering methods, the three-cluster solution had highest agreement (84.41%), relative to the two and four cluster solutions (78.34%, 58.70%, respectively), and far exceeded the 25% greater than chance threshold. Using K-means as the standard, there was agreement on 89.61% (*n* = 138) of the Cognitively Average group, 83.36% (*n* = 189) of the Low EF group, and 79.67% (*n* = 90) of the Prominently Impaired EF/Memory group. There was a significant relationship between the likelihood of being assigned to the equivalent group using both clustering methods (χ^2^(4) = 598.73, *p* < 0.001). Thus, the three K-means clusters were determined to be the optimal cluster solution and were used in all subsequent analyses.

Table 3 depicts the demographic, cognitive, mood, and motor scores of the three cognitive clusters. Results of Kruskal–Wallis H tests indicated that the three clusters significantly differed across DRS-2 total scores and all cognitive composite scores in the expected direction. Specifically, participants in the Cognitively Average group performed better than those in the Low EF group, who performed better than those in the Prominently Impaired EF/Memory group across all cognitive indices.

As shown in Table 3, the three clusters also differed with respect to motor symptom severity, self-reported mood symptoms, and racial/ethnic distribution. Namely, participants in the Prominently Impaired EF/Memory cluster had significantly greater motor symptoms (UPDRS Part III scores), greater apathy (AS), and greater trait anxiety (STAI: Trait) than both other clusters and greater depressive symptoms (BDI-II) than the Cognitively Average cluster. Those in the Low EF and Prominently Impaired EF/Memory clusters had significantly greater state anxiety (STAI: State) than the Cognitively Average cluster. Moreover, there was a significant difference between the clusters’ proportions of white non-Hispanic participants, with a fewer represented in the Cognitively Average cluster relative to both other clusters. In contrast, there were no significant differences among the clusters across other demographic characteristics (age, education, sex), duration of illness (years since diagnosis, years since symptom onset), or proportion of participants with each motor subtype. Overall, the Prominently Impaired EF/Memory cluster had significantly worse mood, motivation, and motor symptoms than the other two clusters, but the effect sizes of these differences were small. As a follow-up, we conducted Pearson correlation analyses between the cognitive composites and mood/motivation measures (See Table A2 for analyses). Overall, the results continued to suggest an exceptionally small but consistent relationship between greater mood symptoms and worse cognitive performance across domains. Additionally, we conducted exploratory analyses within the language domain to determine whether one of the two measures drove the low language performance seen within the Prominently Impaired EF/ Memory cluster. We found that semantic fluency performance was significantly lower than confrontation naming (see Table A3).

### 3.4. Relationship between Cognitive Phenotypes and PD-MCI Classification

Table 4 depicts the distribution of PD-MCI cases within each cluster using the three PD-MCI impairment cutoffs. Pearson chi-square tests of independence revealed that PD-MCI classification and cluster membership were significantly related to one another with a large effect size (Table 4). Across all three cutoffs, the Cognitively Average cluster contained very few PD-MCI cases. Using the −1 SD cutoff, the Low EF and Prominently Impaired EF/Memory clusters contained similar portions of the PD-MCI cases. Using the −1.5 SD cutoff, about three quarters of the PD-MCI cases fell into the Prominently Impaired EF/Memory cluster while about a quarter fell into the Low EF cluster. Finally, using the −2 SD cutoff, almost all the PD-MCI cases fell into the Prominently Impaired EF/Memory cluster. Thus, the more stringent the impairment cutoff, the more sensitive the Prominently Impaired EF/Memory cluster was to containing greater portions of the PD-MCI classified cases.

Binary logistic regression analyses examined how well cluster membership predicted PD-MCI classification. Each impairment cutoff had significant omnibus tests (−1 SD: X^2^(2) = 274.61, *p* < 0.001, Cox and Snell *R*^2^ = 0.43, Nagelkerke *R*^2^ = 0.58; −1.5 SD: X^2^(2) = 203.37, *p* < 0.001, Cox and Snell *R*^2^ = 0.34, Nagelkerke *R*^2^ = 0.52; −2 SD: X^2^(2) = 128.35, *p* < 0.001, Cox and Snell *R*^2^ = 0.23, Nagelkerke *R*^2^ = 0.50). The models all had large effect sizes, explaining between 50–58% of the variance in PD-MCI classification, and cluster membership was a significant predictor in all three impairment cutoff models.

Using the −1 SD cutoff, those in the Cognitively Average cluster had 1000× lower odds of being classified as PD-MCI, relative to the Prominently Impaired EF/Memory cluster, while those in the Low EF cluster had 16.95× lower odds (*p*’s < 0.001; Cognitively Average cluster’s 95% Confidence Interval (CI): 200-perfect model fit; Low EF’s 95% CI: 8.29–35.71). This model correctly classified 79.1% of PD-MCI cases with poor sensitivity (as only about half of PD-MCI cases were classified as such by the model) and excellent specificity (as almost all Cognitively Average cluster participants were correctly classified as cognitively normal by the model; Table 5). Using the −1.5 SD cutoff, those in the Cognitively Average cluster had 333.33× lower odds of being classified as PD-MCI, relative to the Prominently Impaired EF/Memory cluster, while those in the Low EF cluster had 18.87× lower odds (*p*’s < 0.001; Cognitively Average’s 95% CI: 47.62-perfect model fit; Low EF’s 95% CI: 10.53–33.33). This model correctly classified 87.9% of PD-MCI cases with stronger sensitivity and slightly lower (but still excellent) specificity than the −1 SD criteria model. Finally, using the −2 SD cutoff, those in the Low EF cluster had 71.43x lower odds of being classified as PD-MCI, relative to the Prominently Impaired EF/Memory cluster, while no cases in the Cognitively Average cluster were classified as PD-MCI (Low EF *p* < 0.001, 95% CI: 16.39–333.33). This final model correctly classified 90.9% of PD-MCI cases, but the model predicted that all cases were cognitively normal, leading to a null sensitivity and perfect specificity.

When using the models’ predicted PD-MCI group membership to predict actual PD-MCI classification, all three models produce significant, acceptable C statistics (above 70%, *p*’s < 0.001; Table 5). The model using the −1.5 SD cutoff had the highest jointly maximized sensitivity and specificity (−1 SD Youden’s Index (YI): 0.50; −1.5 SD YI: 0.66; −2 SD YI: 0). Table 5 presents the positive and negative predictive values predicated on a range of PD-MCI base rate estimates. Using the sample’s prevalence rates for each cutoff, the −1 SD cutoff maximizes the probability that those classified by the model as PD-MCI truly meet this actuarial diagnosis while the −1.5 SD cutoff maximizes the probability that those classified as cognitively normal are truly cognitively intact. Since the true base rate of PD-MCI is unknown, the low, midpoint, and high end of the range of estimated prevalence rates were also used to calculate positive and negative predictive values [25,26]. In settings with lower PD-MCI prevalence rates (e.g., 25%), using −1 SD would jointly maximize the probability of correct classification, but in settings containing a population with greater chance of impairment (e.g., 45%), then −1.5 SD would do so. As our patients were all seen in a specialty clinic, the prevalence of PD-MCI in our sample is presumably closer to this midpoint of the prevalence rates previously estimated. Taken all together, using the −1.5 SD cutoff resulted in cluster membership having high model-based classification accuracy, the largest YI, and jointly maximized PPV/NPV (based on our sample’s clinical setting).

## 4. Discussion

Our study investigated different techniques of methodologically defining and characterizing cognitive impairment in a large, clinical sample of individuals with idiopathic PD without dementia. We took two approaches (i.e., actuarial PD-MCI classification, cluster analytic) and looked at their overlap. In doing so, we hoped to learn which cognitive phenotypes empirically emerged, the influence of different impairment cutoffs on PD-MCI prevalence rates, and whether a specific impairment cutoff aligned best with the cognitive phenotype reflecting greater PDD risk.

The actuarially defined PD-MCI prevalence varied from 40.1% using the liberal end of impairment criteria (−1 SD), to 21.5% using the midpoint (−1.5 SD), and 9.1% using the conservative end (−2 SD). Regardless of impairment cutoff, most PD-MCI cases involved single-domain impairment. This finding aligns with that of Marras and colleagues [62] and runs counter to that of others who describe PD-MCI as most commonly involving multi-domain impairment [25,62,63]. This discrepancy likely reflects methodological differences. For example, if one strictly follows proposed criteria for MCI by the Movement Disorder Society (MDS) [23], then having a single impaired test across two separate domains leads to the designation of multi-domain PD-MCI. In contrast, other more psychometrically rigorous approaches require participants to have at least two impaired measures within a domain for it to be considered impaired. Marras and colleagues [62] found that when strictly using MDS criteria, most PD-MCI cases demonstrated multi-domain impairment; however, when they analyzed the same sample with an altered operationalization of impairment that required two impaired tests *within* a domain, the majority of PD-MCI cases had single domain impairment. Thus, using a similarly more stringent operationalization of impairment, our results support the commonality of the single-domain impairment in a larger sample of individuals with PD without dementia.

In the current study, we grouped those classified as PD-MCI into executive vs. nonexecutive subtypes, with presence or absence of other co-occurring cognitive difficulties (e.g., EF—single domain, EF—multi-domain). This method of subtyping allowed us to distinguish the variety of cognitive domains impaired beyond executive dysfunction. Using this approach, we found, across all three cutoffs, the Single-EF subtype was the most prevalent, but 19–26% of PD-MCI cases (depending on the cutoff) exhibited impairments only in other domains-reinforcing the existence of variable patterns in cognitive performance across individuals with PD. Beyond the most prevalent deficit, executive dysfunction, participants primarily demonstrated deficits in memory and language domains.

Using a different statistical approach (cluster analysis), we found three distinct cognitive phenotypes in this same sample of individuals with PD. These phenotypes differed in severity of cognitive deficits: (1) average cognition, (2) low EF, and (3) more prominent impairments in EF and memory with low language abilities. Though this is a cross-sectional study, it is possible that these phenotypes may reflect the succession of neuropathological changes. Namely, individuals with PD develop frontal executive dysfunction based on dopaminergic changes in frontal-striatal networks and, as the disease progresses, more cortical system and limbic deficits develop (based on cholinergic changes) [29,30,31]. With disease progression, individuals with greater cognitive deficits may have more widespread involvement of cortical areas which likely increases their risk of transitioning to PDD. This notion was recently supported by Domellöf and colleagues [64] who found that significantly lower performance on semantic fluency, memory, and EF measures, identified individuals with PD-MCI who converted to PDD over a five-year period. Thus, our broadly impaired cluster contained multiple deficits previously shown to be predictive of progression to PDD.

Participants in the more prominently impaired cluster demonstrated worse motor symptoms (UPDRS-Part III scores) than the other two clusters. This finding is in alignment with previous research showing greater cognitive dysfunction (including dementia risk) in those with more advanced motor symptoms and disease progression [65,66,67]. The prominently impaired cluster also had greater mood symptoms relative to the other two clusters. While the influence of mood is likely a contributing component to their performance, the cluster differences had small effect sizes, suggesting a minimal impact of mood on cluster membership. We did not find broad evidence for demographic differences between the three clusters, a finding that conflicts with prior research that older age, less education, and longer disease duration are associated with worse cognition [68,69,70]. Of note, the cognitive clusters were based on composite scores of “normed” neuropsychological measures, which controls for certain demographic factors. As such, the norming might obfuscate findings of age, and potentially education, differences across the cluster phenotypes. We did find that the cognitively average cluster had a greater proportion of white non-Hispanic participants; however, the small sample sizes of other races/ethnicities in the overall sample limit our ability to draw related inferences.

Finally, the outcomes of the data-driven and theoretical approaches were compared. We found that the midpoint cutoff (−1.5 SD) served as the best predictor of PD-MCI classification relative to the other cutoffs in terms of classification accuracy and jointly maximized sensitivity and specificity of the model. Use of the midpoint (−1.5) cutoff resulted in three-fourths of the PD-MCI cases falling into the prominently impaired cluster, whereas use of liberal (−1 SD) cutoff resulted equivalent distribution of PD-MCI cases into the low EF and prominently impaired clusters. Researchers previously tried to validate the same three PD-MCI impairment cutoffs by comparing them against clinical diagnosis, but findings have been inconsistent, supporting cutoffs at either −1.5 SD [71] or −2 SD [8]. Furthermore, these clinical validation methods use a circular logic in that clinical diagnosis of impairment would still require a preconceived understanding of what performance counts as impaired relative to a normative expectation. Comparing actuarial and empirical approaches serves as a validation method that circumvents this circular logic. This methodology has precedence within the Alzheimer’s literature which found that data-driven approaches may enhance sensitivity for detecting both cross-sectional and future clinical, biomarker, and neuropathological outcomes-a topic that should be explored in future work with PD participants.

Past longitudinal work evaluating which impairment cutoff best predicts PDD development over a multi-year follow-up period also remains mixed-with some studies supporting an optimal cutoff at −1.5 SD [27] while others support a cutoff of −2 SD [72]. Though cross-sectional, our findings further support the use of a midpoint (−1.5 SD) cutoff based on its validation against the sample’s cognitive phenotype with deficits that past studies suggest are predictive of PDD.

This study has limitations. First, our clinical sample may not be representative of a broader population of individuals with PD as our data were collected at a specialty center from a combination of patients seeking deep brain stimulation and those referred for a neuropsychological evaluation due to cognitive concerns. Second, while patients were clinically diagnosed with PD using established clinical criteria, this patient population did not have pathologically confirmed PD (to definitively rule out other syndromes that could mirror PD early on, such as Progressive Supranuclear Palsy-Parkinsonism Predominant variant). Third, we did not have access to the whole sample’s medication information that would enable us to characterize the potential relationship of cognitive performance with medications (e.g., parkinsonian medication’s LEDD values, or rate of anticholinergic use). A small subset of recently seen patients (*n* = 20/cluster) had available medication lists, from which Magellan Anticholinergic Risk Scale scores were calculated [73]. A chi-square test of independence showed no significant differences in the proportions of Magellan scores across clusters and weak effect size (*p* = 0.516, Cramer’s V = 0.20), suggesting that our predominantly impaired group did not have a significantly disproportionate amount of participants on anticholinergic medication. Fourth, we used listwise exclusion to meet the goal that all participants have two or more measures within each domain to classify them as PD-MCI. There is a possibility that we may have missed more severely impaired individuals who did not complete the full battery of tests, though these individuals would have likely been excluded based on our cognitive screener.

More broadly, there are some issues involving current practices used for formal clinical diagnosis of MCI. One practice is the requirement that patients have subjective cognitive complaints in order to receive MCI diagnosis (as in the MDS PD-MCI consensus criteria). We did not have this information readily available, and not applying this broadly used MDS criteria limits the comparability of our findings to other studies that use it. Even so, there is precedence for not including subjective cognitive complaints within an actuarial version of the PD-MCI criteria [7,74,75,76], and evidence for the usefulness of this criteria is mixed [77,78]. Finally, clinicians often make a diagnosis of MCI in situations where there has been a performance drop from an estimated premorbid baseline (e.g., a, drop from superior to low average ranges). We were unable to account for impairment based on decline from premorbid abilities in this sample. However, defining MCI with actuarial methods has gained traction within the Alzheimer’s literature and been shown to improve diagnostic rigor for MCI [22,79,80,81]. Thus, further research examining actuarial methods to characterize PD-MCI may prove helpful in establishing a “gold standard” and “true” prevalence rates.

Future work should expand to a more racially/ethnically diverse sample with wider levels of educational attainment to better generalize the results to a broader patient population. It would also be helpful to conduct a longitudinal study to evaluate which impairment cutoff best predicts PDD development in a larger sample than previous studies.

## 5. Conclusions

The current findings add to the literature by demonstrating the utility of comparing empirical and classification definitions of cognitive impairment. Our results reinforce that variability in prevalence rates of actuarially-defined PD-MCI stems, in part, from use of different normative definitions of impairment (e.g., −1 to −2 SD). Although this may seem trivial, it dramatically affects prevalence rates and in turn influences predictive validity of dementia. Yet, across all cutoffs, PD-MCI classified cases most commonly exhibited single-domain deficits (primarily in executive function). When empirically defining patterns of cognitive impairment in a large clinical sample, we found three distinct cognitive phenotypes with differing levels of cognitive deficit severity. The cognitive phenotype with broader, more prominent impairments (including features suggestive of greater risk of impending PDD development) best aligned with operationalizing impairment at the midpoint cutoff (−1.5 SD). These findings contribute to the widespread efforts to determine criteria that best establish what level and which patterns of cognitive impairment have the most utility at predicting who is at greatest risk of upcoming progression to PDD.

## Figures and Tables

**Table 1:** Neuropsychological Tests within Each Cognitive Domain Composite.

Cognitive Domain	Tests	Raw Score Used
**Executive Functioning**	Stroop Test (Interference trial) TMT Part B Letter Fluency (FAS)	Total Number of Correct Items Completion Time Total Number of Words (all 3 trials)
**Verbal Delayed Memory**	HVLT-R WMS-III Logical Memory	Delayed Total Recall Delayed Total Recall
**Language**	BNT Semantic Fluency (Animals) *	Total Correct Spontaneous Responses Total Number of Words
**Visuospatial Skills**	Benton JOLO Benton FRT	Total Items Correct Total Items Correct
**Attention/Working Memory**	WAIS-III Digit Span Forward WAIS-III Digit Span Backward	Total Number of Points Total Number of Points

Note. * While controversy remains over which cognitive domain should include semantic fluency, inclusion within the Language domain has precedent within other PD-MCI studies [42,43]. Stroop Test is the Golden version [44]; TMT Part B = Trails Making Test Part B [45]; Letter Fluency (FAS) [46]; HVLT-R = Hopkin’s Verbal Learning Test-Revised [47]; WMS-III = Wechsler Memory Scale-Version III [48]; BNT = Boston Naming Test [49]; Semantic Fluency (Animals) [50]; Benton JOLO = Benton Judgment of Line Orientation [51]; Benton FRT = Facial Recognition Test [52]; WAIS-III = Wechsler Adult Intelligence Scale-Version III [53].

**Table 2:** Sample Descriptive Characteristics.

Measure	Overall Sample (*n* = 494)
*Variable*	*Mean/% (SD)*
Age	64.73 (9.04)
Education (years)	15.01 (2.79)
% Male	72%
% White, non-Hispanic	94%
Years since diagnosis	7.84 (4.94)
Years since symptom onset	9.61 (5.26)
PD motor subtype	
Tremor predominant	77%
Akinetic-rigid	22%
PIGD	1%
UPDRS III, on medication	25.28 (9.80)
Hoehn and Yahr (H-Y) Scale ^^^	
0	0.30%
1	1%
1.5	1%
2	58%
2.5	21%
3	15%
4	3%
BDI-II, raw total	10.10 (6.86)
STAI: State anxiety, percentile	61.33 (29.87)
STAI: Trait anxiety, percentile	58.38 (30.73)
Apathy scale, raw total	11.22 (6.31)
Dementia Rating Scale-2, raw total	136.99 (4.49)
Cognitive composites (z-scores) ^#^	
Executive function	−0.58 (0.90)
Verbal delayed memory	−0.36 (1.01)
Language	−0.03 (0.96)
Visuospatial abilities	0.01 (0.78)
Attention/working memory	0.18 (0.77)

Note. UPDRS = Unified Parkinson’s Disease Rating Scale; PIGD = Postural Instability–Gait Difficulty; BDI-II = Beck Depression Inventory-II, STAI = State-Trait Anxiety Inventory; ^^^ = H-Y scores available for 78% of the sample (*n* = 389); ^#^ *z*-score has a mean of 0 and SD of 1, and z-score composites were computed from performance on neuropsychological tasks within a domain.

**Table 3:** Comparing the K-means Clusters’ Descriptive, Clinical, and Cognitive Characteristics.

Characteristic Measure	Cluster 1 Cognitively Average	Cluster 2 Low EF	Cluster 3 Prominently Impaired EF/Memory	Omnibus Kruskal– Wallis H-Test	Effect Size	Post hoc Differences (Bonferroni Corrected)
	*N* = 154	*N* = 227	*N* = 113			
	Mean (SD)/	Mean (SD)/	Mean (SD)/	*p-*value	Eta squared /Cramer’s V *^#^*	
	%	%	%			
Age (years)	64.51 (8.75)	65.31 (9.12)	63.85 (9.15)	0.38	0.004	--
Education (years)	15.35 (2.55)	14.91 (2.91)	14.73 (2.83)	0.13	0.01	--
Sex (% Male)	68%	73%	75%	0.33 ^^^	0.07 ^#^	--
% Caucasian, Non	99%	93%	91%	0.01 ^^^	0.15 ^#^	1 < [2 = 3] *
Hispanic						
% H-Y Stages 2–3	95%	93%	95%	0.57 ^^^	0.06 ^#^	-
% Tremor Subtype	73%	76%	82%	0.16 ^^^	0.08 ^#^	--
Years Since Symptom	9.45 (5.14)	9.51 (5.20)	10.03 (5.57)	0.58	0.002	--
Onset						
Years Since Diagnosis	7.70 (5.06)	7.58 (4.73)	8.56 (5.15)	0.23	0.01	--
UPDRS Part III	22.78 (8.85)	25.18 (9.38)	28.91 (10.81)	<0.001	0.05	[1 = 2] < 3 **
BDI-II	9.15 (7.04)	9.81 (6.06)	12.09 (7.79)	0.001	0.03	1 < 3 **
Apathy Scale	10.47 (6.33)	10.89 (6.25)	13.01 (6.14)	0.01	0.02	[1 = 2] < 3 **
STAI: State Pct.	53.20 (31.34)	63.26 (28.51)	68.73 (28.09)	<0.001	0.04	1 < [2 = 3] **
STAI: Trait Pct.	50.11 (31.29)	59.21 (30.30)	68.33 (27.73)	<0.001	0.04	1 < 2 < 3 **
DRS-2	139.54 (3.10)	136.92 (4.22)	133.65 (4.42)	<0.001	0.23	1 > 2 > 3 *
Cognitive Domain						
*Z*-Score Composites						
Executive Function	0.20 (0.59)	−0.58 (0.58)	−1.63 (0.65)	<0.001	0.56	1 > 2 > 3 *
Memory	0.51 (0.70)	−0.44 (0.75)	−1.40 (0.75)	<0.001	0.47	1 > 2 > 3 *
Language	0.79 (0.68)	−0.08 (0.70)	−1.02 (0.72)	<0.001	0.48	1 > 2 > 3 *
Visuospatial Skills	0.44 (0.56)	0.01 (0.70)	−0.59 (0.82)	<0.001	0.21	1 > 2 > 3 *
Attention/WM	0.67 (0.70)	0.03 (0.72)	−0.20 (0.63)	<0.001	0.20	1 > 2 > 3 **

Note. * Significant difference at *p* < 0.001 across all group comparisons; ** Significant difference at *p* < 0.05 across all group comparisons; ^^^ Chi-square test of independence used; ^#^ Cramer’s V values; EF = Executive Function; WM = Working Memory; H-Y = Hoehn and Yahr; UPDRS = Unified Parkinson’s Disease Rating Scale; BDI-II = Beck Depression Inventory-II, STAI = State-Trait Anxiety Inventory; DRS-2 = Dementia Rating Scale-2.

**Table 4:** Percentage of PD-MCI Cases Falling to Each K-Means Cluster.

Impairment Cutoff	Cognitively Average % (*n*)	Low EF % (*n*)	Prominently Impaired EF/Memory % (*n*)	Pearson Chi Square	*p*-Value	Cramer’s V
−1 SD	1.01% (2)	46.46% (92)	52.53% (194)	223.47	<0.001	0.67
−1.5 SD	0.95% (1)	23.81% (25)	75.24% (79)	213.13	<0.001	0.66
−2 SD	0% (0)	4.44% (2)	95.56% (43)	148.33	<0.001	0.55

Note. PD-MCI = Parkinson’s Disease-Mild Cognitive Impairment; EF = Executive Function.

**Table 5:** Binary logistic regression models’ sensitivity, specificity, and positive and negative predictive values based on different PD-MCI prevalence rates.

Impairment Cutoff	Sensitivity	Specificity	C Stat.	Sample		0.25		Base Rate 0.45		0.65	
				PPV NPV		PPV NPV		PPV NPV		PPV NPV	
−1 SD	0.53	0.97	0.86	0.92	0.76	0.85	0.86	0.94	0.72	0.97	0.53
−1.5 SD	0.75	0.91	0.88	0.70	0.93	0.74	0.92	0.87	0.82	0.94	0.66
−2 SD	0.00	1.00	0.91	--	0.91	--	0.75	--	0.55	--	0.35

Note. PD-MCI = Parkinson’s Disease–Mild Cognitive Impairment; C Stat. = C Statistic; PPV = positive predictive value; NPV = negative predictive value.

## Data Availability

The datasets presented in this article are not publicly available because an inquiring party must submit a request to the UF INFORM database committee at the Norman Fixel Institute of Neurologic Diseases, and this request must be approved by the UF IRB. Requests to access the datasets should be directed to “Chuck Jacobson, jacobson@neurology.ufl.edu”.

## Appendix A. Comparison of Deep Brain Stimulation and General Clinic Patients’ Cognitive Performance

**Table A1:** Cognitive performance of deep brain stimulation candidates versus general clinic patients.

Measure	DBS	General Clinic	Significance
	(*n* = 338)	(*n* = 156)	
	*Mean (SD)*	*Means (SD)*	*p-value*
Dementia Rating Scale-2, raw total	137.05 (4.28)	136.86 (4.92)	0.69
Cognitive Composites (*z*-scores) ^#^			
Executive Function	−0.55 (0.88)	−0.64 (0.93)	0.32
Verbal Delayed Memory	−0.37 (1.00)	−0.36 (1.04)	0.95
Language	0.001 (0.94)	−0.09 (1.00)	0.35
Visuospatial Abilities	0.01 (0.78)	0.02 (0.79)	0.85
Attention/Working Memory	0.20 (0.77)	0.11 (0.76)	0.22

Note. ^#^ *z*-score has a mean of 0 and SD of 1, and *z*-score composites were computed from performance on neuropsychological tasks within a domain. Bootstrapped independent sample *t*-tests were performed to assess group differences.

## Appendix B. Relationship between Mood and Cognitive Domains

**Table A2:** Pearson correlations between mood measures and composite *z*-scores.

Measure	Executive Function	Verbal Delayed Memory	Language	Visuospatial	Attention/ Working Memory
BDI-II	−0.10 *	−0.13 *	−0.09 *	−0.10 *	−0.16 *
STAI					
State	−0.18 *	−0.10 *	−0.18 *	−0.10 *	−0.08
Trait	−0.18 *	−0.15 *	−0.17 *	−0.14 *	−0.15 *
Apathy Scale	−0.14 *	−0.10	−0.09	−0.13 *	−0.09

Note. * *p* < 0.05.

Greater depressive symptoms (BDI-II) and dispositional anxiety (STAI-Trait) were both significantly associated with poorer performance across all composites, with small effect sizes. Greater situational anxiety (STAI-State) significantly related to worse performance on all composites except attention/working memory, with small effect sizes, and greater apathy symptoms (AS) significantly correlated with poorer performance on executive and visuospatial composites.

## Appendix C. Exploratory Analyses of Language Metrics

**Table A3:** *Z*-scores of Language Metrics across Clusters.

Metric	Cognitively Average	Low EF	Prominently Impaired EF/Memory
Boston Naming Test	1.05 (0.96)	0.18 (1.01)	−0.67 (0.96)
Semantic Fluency (Animals)	0.52 (0.90)	−0.35 (0.96)	−1.37 (1.04)

Note. Values are presented as normative *z*-score mean (standard deviation).

Table A3 shows mean scores of BNT and semantic fluency (Animals) across the 3 clusters. Results of a between (cluster) x within (language measure) repeated measures ANOVA revealed a significant main effect of language measures (*F*(1.00, 491.00) = 95.94, *p* < 0.001, *n_p_*^2^ = 0.16) where semantic fluency was significantly lower than confrontation naming (M = −0.65, *p* < 0.001). The main effect of cluster was also significant (*F*(2, 433.61) = 202.84, *p* < 0.001), with the Cognitively Average cluster performing significantly better than the Low EF cluster (M difference = 0.87) who in turn scored better than the Prominently Impaired EF/Memory cluster (M difference = 0.78), with all *p* values < 0.001. The cluster x language measure interaction was nonsignificant (*F*(2.00, 491.00) = 0.50, *p* > 0.05, *n_p_*^2^ = 0.002). Thus, all clusters demonstrated similar patterns of worse semantic fluency performance relative to confrontation naming.
